# Transglutaminase 2 as an independent prognostic marker for survival of patients with non-adenocarcinoma subtype of non-small cell lung cancer

**DOI:** 10.1186/1476-4598-10-119

**Published:** 2011-09-24

**Authors:** Chang-Min Choi, Se-Jin Jang, Seong-Yeol Park, Yong-Bock Choi, Jae-Heon Jeong, Dae-Seok Kim, Hyun-Kyoung Kim, Kang-Seo Park, Byung-Ho Nam, Hyeong-Ryul Kim, Korean Thoracic Oncology Research Group (KTORG), Soo-Youl Kim, Kyeong-Man Hong

**Affiliations:** 1 Department of Pulmonary and Critical Care Medicine, Asan Medical Center, College of Medicine, University of Ulsan, 388-1 Pungnap-2 Dong, SongPa-Gu, Seoul 138-736, Korea; 2 Department of Pathology, Asan Medical Center, College of Medicine, University of Ulsan, 388-1 Pungnap-2 Dong, SongPa-Gu, Seoul 138-736, Korea; 3 Cancer Cell and Molecular Biology Branch, Research Institute, National Cancer Center, 323 Ilsan-ro, Ilsandong-gu, Goyang 410-769, Korea; 4 Department of Medical Oncology & Hematology, Kyung Hee Medical Center, Kyung Hee University, Seoul 130-702, Korea; 5 Cancer Biostatics Branch, Research Institute, National Cancer Center, 323 Ilsan-ro, Ilsandong-gu, Goyang 410-769, Korea; 6 Department of Thoracic and Cardiovascular Surgery, Asan Medical Center, College of Medicine, University of Ulsan, 388-1 Pungnap-2 Dong, SongPa-Gu, Seoul 138-736, Korea

## Abstract

**Background:**

Expression of transglutaminase 2 (TGase 2) is related to invasion and resistance to chemotherapeutic agents in several cancer cells. However, there has been only limited clinical validation of TGase 2 as an independent prognostic marker in cancer.

**Methods:**

The significance of TGase 2 expression as an invasive/migratory factor was addressed by *in vitro *assays employing down-regulation of TGase 2. TGase 2 expression as a prognostic indicator was assessed in 429 Korean patients with early-stage non-small cell lung cancer (NSCLC) by immunohistochemical staining.

**Results:**

TGase 2 expression increased the invasive and migratory properties of NSCLC cells *in vitro*, which might be related to the induction of MMP-9. In the analysis of the immunohistochemical staining, TGase 2 expression in tumors was significantly correlated with recurrence in NSCLC (p = 0.005) or in the non-adenocarcinoma subtype (p = 0.031). Additionally, a multivariate analysis also showed a significant correlation between strong TGase 2 expression and shorter disease-free survival (DFS) in NSCLC (p = 0.029 and HR = 1.554) and in the non-adenocarcinoma subtype (p = 0.030 and HR = 2.184). However, the correlation in the adenocarcinoma subtype was not significant.

**Conclusions:**

TGase 2 expression was significantly correlated with recurrence and shorter DFS in NSCLC, especially in the non-adenocarcinoma subtype including squamous cell carcinoma.

## Background

Lung cancer is the leading cause of cancer-related death, accounting for approximately 29% of all cases (*Cancer Stat Fact Sheets*, http://www.seer.cancer.gov); approximately 85% of lung cancer cases are non-small cell lung cancer (NSCLC). There are several different subtypes of NSCLC, among which are adenocarcinoma and squamous cell cancer. Currently, the NSCLC subtypes are regarded as a single disease, however, the adenocarcinoma and non-adenocarcinoma subtypes are regarded as being separate entities, owing to their different responses to recently developed agents such as pemetrexed, gefitinib, bevaciuzumab, and crizotinib, which are more effective in adenocarcinoma [1-3]. Accordingly, identification of the molecular differences between these tumor types will have a significant impact on the design of novel therapies that can improve treatment outcomes.

Transglutaminase 2 (TGase 2) is a multifunctional protein that can bind and hydrolyze GTP as well as catalyze covalent cross-links [4]. The biological role of TGase 2 in the development of resistance to cisplatin and doxorubicin in several cancer cells has drawn considerable attention [5-9]. Another biological role of TGase 2, this one in cancer metastasis and invasion, was reported for breast, pancreatic, and ovarian cancers [10-13]. However, the role of TGase 2 expression as an independent prognostic factor has not been well elucidated, except for a study on ovarian cancer [11]. Moreover, the possibility that TGase 2 has different roles in different subtypes of any cancer has never been suggested. Accordingly, in the present study, after first testing the biological role of TGase 2 in invasion and migration with NSCLC cell lines, its role as a prognostic indicator in NSCLC was investigated in an immunohistochemical study on early-stage NSCLC tissues.

## Materials and methods

### Cell lines

Human squamous lung cancer cell lines H1703 and HCC-95 were obtained from the Korean Cell Line Bank and maintained in RPMI 1640 supplemented with 10% fetal bovine serum (Thermo Fisher Scientific Hyclone, Logan, UT), 1 mM sodium pyruvate, and 100 U/mL penicillin-streptomycin at 37°C in a humidified 5% CO_2 _incubator.

### Scratch cell migration assay

Parental cells or cells transfected with control or small interfering RNAs (siRNAs) targeting TGase 2 were grown to confluence, at which time they were scratched with a pipette tip. Cell lines not treated with siRNA were starved by incubation overnight in serum-free medium. The cultures were rinsed to remove detached cells and then incubated for 24-48 h. After incubation, the cells were fixed and visualized by light microscopy. These assays were performed three times.

### Matrigel cell invasion assay

The invasive behavior of cells was determined *in vitro *using BioCoat Matrigel Invasion Chamber (BD Biosciences, Bedford, MA) inserts. The cells were trypsinized, and the resulting cell pellets were resuspended in serum-free medium at a final concentration of 1 × 10^5 ^cells, after which 500 μL of suspended cells was added to the insert with 750 μL of complete medium. After 24 or 48 h incubation, the cells that passed through the filter to the underside of the membrane were stained and counted under a light microscope. Ten fields of cells were counted for each well, and the mean number of cells per field was calculated. Each experiment was performed in triplicate and repeated at least two times.

### Transfection

TGase 2 was knocked down by introducing an siRNA duplex targeting human *TGM2 *mRNA (5'-AAG AGC GAG AUG AUC UGG AAC-3') into cells using Fugene 6 (Roche, Mannheim, Germany) according to the manufacturer's instructions. Briefly, cells were seeded at a density of 3 × 10^5 ^per well in a six-well plate. When the cells reached about 60% confluence, 400 μL of an siRNA-Fugene 6 mixture was added, and the cells were incubated for 48 h in a 5%CO_2 _incubator.

### Western blot analysis

Equal amounts of each cell lysate were electrophoresed on sodium dodecyl sulfate-polyacrylamide gels, after which resolved proteins were transferred to polyvinylidene difluoride membranes. The membranes were incubated with various primary antibodies diluted in TBST (20 mM Tris, 134 mM NaCl, 0.02% Tween 20). The primary antibodies were then detected with horseradish peroxidase-conjugated secondary antibodies followed by exposure to an enhanced chemiluminescence reagent. The antibodies for MMP-9, MMP-2 and Vimentin were purchased from Santa Cruz Biotechnology (Santa Cruz, California); those for E-cadherin and β-actin were obtained from Abcam (Cambridge, MA).

### Gelatin zymography

MMP-9 activity in the cultured cells was measured using gelatin-gel zymography. The ten-fold-concentrated culture medium was electrophoretically resolved by 7.5% SDS-PAGE containing 0.1% gelatin under the non-reducing condition. The gel was washed in 2.5% Triton X-100 for 30 min to remove SDS and incubated overnight at 37°C in 50 mM Tris-Cl, pH 7.5, and 5 mM CaCl_2_. After Coomassie blue staining and destaining of the gel, the MMP-9 activity was detected as a clear band on a dark blue background.

### NSCLC cancer tissues and other human tissues

A total of 429 NSCLC cases for which surgery had been performed at the Asan Medical Center (Seoul, Korea) between 2000 and 2003 were selected from the archives of the Department of Pathology, and tissue arrays were prepared. The use of relevant human archival tissues was approved by the Institutional Review Board. All available histological slides, which had been routinely stained with hematoxylin and eosin (H&E), were reviewed. As controls, a variety of human tissue array sections (BB6, CCA3, CC4, CSA3, CBA3 and CDA2), purchased from SuperBioChip Laboratories (http://www.tissue-array.com), were used.

### Immunohistochemical staining of TGase 2 in NSCLC

Immunohistochemical staining was performed using an Ultravision LP Detection kit (Lab Vision, USA). The tissue sections were deparaffinized with xylene, and antigen retrieval was accomplished by autoclaving the slides in 10 mM Tris (pH 9.0) and 1 mM EDTA for 15 min. After fixing in 95% ethanol, the slides were treated with 3% hydrogen peroxide, incubated with Ultra V block solution for 15 min, and then incubated with anti-TGase 2 primary antibody (NeoMarkers, Fremont, CA) at a dilution of 1:200 for 1 h at room temperature. After washing in TBST, the slides were treated with primary antibody enhancer for 10 min and then incubated with HRP-conjugated secondary antibody for 15 min. The slides were then washed with TBST, incubated with DAB chromogen, and counterstained with Mayer's Hematoxylin (Dako Cytomation, Glostrup, Denmark). Negative controls, from which the primary antibody had been omitted, were run in parallel. Immunostaining was graded semi-quantitatively, considering both staining intensity and the percentage of positive tumor cells, by two study pathologists blinded to the clinicopathologic variables. The staining intensity was scored on a 0-to-3 scale: 0, no staining of cancer cells; 1, weak staining; 2, moderate staining; 3, strong staining. Also, the percentage of stained tumor cells was scored on a 0-to-3 scale: 0, <10%; 1, 10%-49%; 2, 50%-75%; 3, >75%. The two scores were multiplied, and TGase 2 expression was defined as negative, if the product was 0; intermediate, if the product was 1-3; and strong, if the product was 4 or more.

Expression of TGase 2 was found in cells of normal structure, specifically in smooth muscle and blood vessel endothelial cells. However, there was no such expression in alveolar or bronchial epithelial cells.

### Statistical analysis

The Chi-square test was used to examine differences in TGase 2 immunoreactivity among the groups. The Kaplan-Meier method was employed to estimate survival distributions, and the survival curves were compared using the log-rank test. Values of p < 0.05 were considered statistically significant.

For the purpose of determining associations between TGase 2 over-expression and clinicopathologic variables, a Chi-square or Fisher's exact test was applied. The Cox proportional hazard regression model was employed for univariate and multivariate analyses of the relationship between DFS and clinicopathologic variables or TGase 2 expression. In the multivariate analyses, the variables T-stage, N-stage and histologic type were included as co-variates. In analyses of the non-adenocarcinoma subtype, clinical stage, histologic type and differentiation status were considered as co-variates. All of the statistical analyses were performed using SPSS software (version 18).

## Results

The question of whether TGase 2 expression confers a metastatic phenotype on lung cancer cells was addressed using two NSCLC cell lines: H1703 and HCC-95. TGase 2 was expressed at high levels in the H1703 cells, but was barely detectable in the HCC-95 cells, as determined by Western blot analysis and reverse transcription-polymerase chain reaction (RT-PCR) (Additional File 1, Figure S1A). RT-PCR and Western blot analysis showed that *TGM2 *siRNA reduced TGase 2 expression in the H1703 cells (Additional File 1, Figure S1A). In addition, TGase 2 down-regulation reduced nuclear factor κB (NF-κB) activity in the H1703 cells (Additional File 1, Figure S1B), as we had previously demonstrated for H1299 [9].

**Figure 1:**
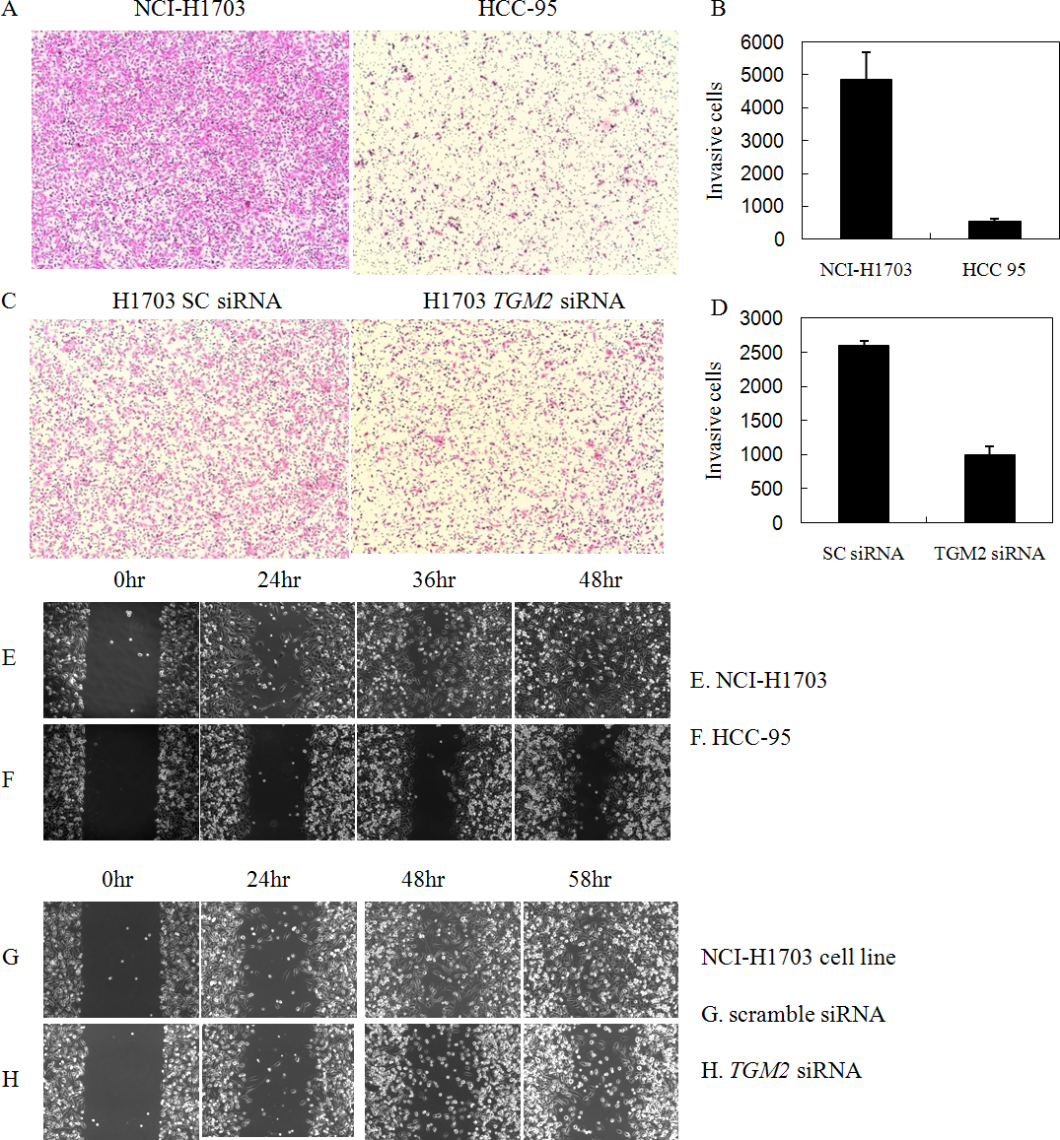
**TGase 2 expression is associated with increased invasion or migration of NSCLC cells**. The levels of invasiveness for the H1703 and HCC-95 cells were compared in a Matrigel invasion assay system (A and B). Representative fields of cells that migrated through the Matrigel to the underside of the membrane were either photographed (A) or counted randomly in 10 fields, with the average numbers of invading cells (±s.d.) plotted (B). Transient transfection of *TGM2 *siRNA decreased the invasiveness for H1703, as shown in the representative fields of cells photographed (C) and the average numbers of invading cells plotted (D). Compared with the HCC-95 cells (E), the cell migration was higher in NCI-H1703 cells having a higher level of TGase 2 (F). The migration was reduced by the treatment of NCI-H1703 with *TGM2 *siRNA and the resultant down-regulation of TGase 2 (G and H). The cell migration status was photographed after the indicated time period.

Because increased cell motility and invasion are important features of metastatic cancer cells, we compared the H1703 and HCC-95 cells for invasiveness, employing a Matrigel invasion assay. As shown in Figures 1A and 1B, the H1703 cells exhibited greater invasiveness than the HCC-95 cells, suggesting that invasiveness might be related to TGase 2-expression levels in NSCLC. To further investigate the role of TGase 2, we transiently transfected H1703 cells with *TGM2 *siRNA to knock down the TGase 2 level. Notably, siRNA-mediated TGase 2 knockdown significantly reduced the invasiveness of the H1703 cells (Figures 1C and 1D). H1703 cells exhibited greater migration than the HCC-95 cells (Figure 1E-1F), and knockdown of TGase 2 by siRNA also reduced the migration of H1703 (Figure 1G-1H).

Further experiments concerning the relationships between TGase 2 expression and MMPs or EMT markers were performed, and it was found that TGase 2 down-regulation by *TGM2 *siRNA is related to down-regulation of MMP-9 but not of MMP-2 (Additional File 2, Figure S2), suggesting that TGase 2's role in invasion and migration might be via the regulation of MMP-9. This is consistent with the previous result for epidermoid carcinoma, a skin cancer [14]. However, in ovarian cancer, MMP-2 rather than MMP-9 regulation by TGase 2 has been reported [13], indicating that different MMPs are regulated by TGase 2 in different types of cancer. The levels of epithelial-mesenchymal transition (EMT) markers such as Vimentin and E-cadherin were not changed by down-regulation of TGase 2 in NCI-H1703 cells (Additional File 2, Figure S2).

**Figure 2:**
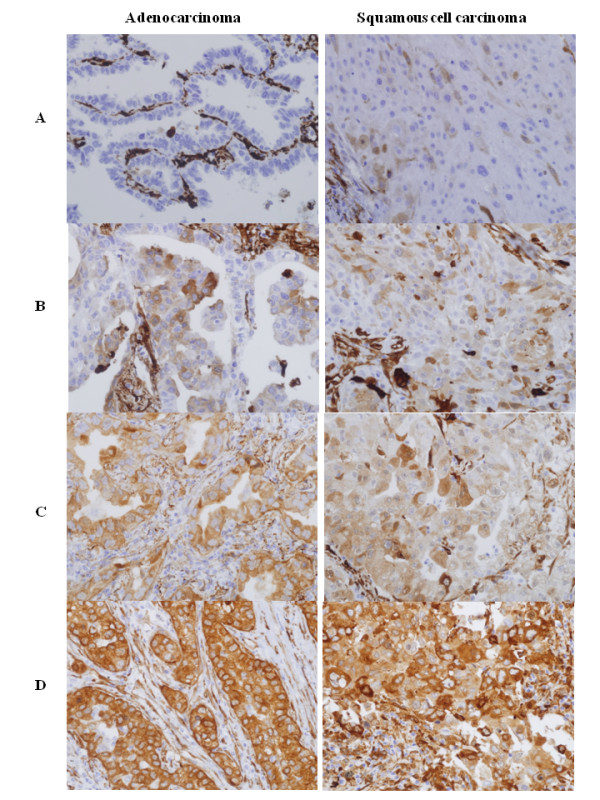
**Representative photomicrographs of TGase 2 immunostaining in arrayed NSCLC tissues**. The left and right columns represent examples of adenocarcinoma and squamous cell carcinoma showing (A) negative, (B) weak, (C) moderate and (D) strong immunoreactivity, respectively. Original magnification: ×400.

To determine the importance of TGase 2 as a prognostic factor in NSCLC cancer patients, we immunostained tissue arrays collected from Korean patients with early-stage NSCLC (Figure 2). Data on the 429 patients are listed in Table 1. All of the patients had undergone curative surgical resection by lobectomy (74.7%), pneumonectomy (16.4%), or bilobectomy (7.5%). About 51.5% of patients were stage I; 23.8% stage II, and 24.7% stage III (Table 1). The median follow-up was 62 months, in a 26-81 month range. The post-resection recurrence rate was about 40%. Histologically, strong TGase 2 expression was also detected in smooth muscle and endothelial cells. Of the 429 NSCLC tissue samples, 93 (21.7%) showed intermediate TGase 2-immunopositivity and 88 (20.5%), strong TGase 2-immunopositivity (Table 2).

**Table 1:** Clinicopathologic characteristics of patients with NSCLC who had undergone complete resection

Variables	No. of subjects (n = 429)
Gender	
Male	324 (75.5%)
Female	105 (24.5%)

Age (median, years)	62 (range: 26-81)

Tumor size (median, cm)	3.8 (range: 0.8-20)

Type of surgical resection*	
Pneumonectomy	70 (16.4%)
Bilobectomy	32 (7.5%)
Lobectomy	319 (74.7%)
Limited resection	6 (1.4%)

Clinical stage	
I	221 (51.5%)
II	102 (23.8%)
III	106 (24.7%)

Adjuvant therapy**	
Chemotherapy only	68 (15.9%)
Radiotherapy only	104 (24.2%)
Neither	184 (42.9%)
Both	71 (16.6%)

Median follow-up period (mean ± SD, months)	65.8(56.3 ± 33.9)

Recurrent cases (recurrence rate)	170 (39.6%)

* Information on the type of surgical resection was not available in two cases.

** Information on radiotherapy was not available in two of the cases treated with chemotherapy.

**Table 2:** Correlation of TGase 2 over-expression with clinicopathologic variables in patients with resectable early-stage NSCLC

Variables	Total number	TGase 2 expression			p-value
		
		Negative	Intermediate	Strong	
Histology					
Adenocarcinoma	193 (45)	74(38)	58(30)	61(32)	**<0.001**
Squamous carcinoma	198 (46)	155(78)	26(13)	17(9)	
Others*	38 (9)	19(50)	9(24)	10(26)	

Differentiation**					
Well	61 (18)	41(67)	11(18)	9(15)	0.474
Moderate	200 (58)	122(61)	41(21)	37(19)	
Poorly	87 (25)	47(54)	18(21)	22(25)	

Gender					
Male	324 (76)	206(64)	58(18)	60(19)	**<0.001**
Female	105 (24)	42(40)	35(33)	28(27)	

Smoking					
Nonsmoker	113 (26)	44(39)	35(31)	34(30)	**<0.001**
Ex-smoker	120 (28)	86(72)	15(13)	19(16)	
Current Smoker	196 (46)	118(60)	43(22)	35(18)	

T-stage***					
1	81 (19)	40(50)	16(20)	25(31)	**0.037**
2	277 (65)	161(58)	67(24)	49(18)	
3-4	70 (16)	47(67)	10(14)	13(19)	

N-stage					
0	257 (60)	148(58)	55(21)	54(21)	0.414
1	93 (22)	53(57)	17(18)	23(25)	
2-3	79 (18)	47(60)	21(27)	11(14)	

Recurrence event					
No	259 (60)	165(64)	52(20)	42(16)	**0.005**
Yes	170 (40)	83(49)	41(24)	46(27)	

Recurrence in male patients					
No	203 (47)	141(69)	33(16)	29(14)	
Yes	121 (28)	65(54)	25(21)	31(26)	**0.011**

Recurrence in female patients					
No	56 (53)	24(42)	19(34)	13(23)	0.672
Yes	49 (47)	18(37)	16(33)	15(31)	

Recurrence in patients with adenocarcinoma					
No	102 (53)	43(42)	29(28)	30(29)	0.512
Yes	91 (47)	31(34)	29(32)	31(34)	

Recurrence in patients with non-adenocarcinoma					
No	157 (67)	122(78)	23(15)	12(8)	**0.031**
Yes	79 (33)	52(66)	12(15)	15(19)	

The significance was tested with Chi-square or Fisher's exact tests (p). The percentages for the variables are shown within parentheses.

*Large cell lung cancer (13), adenosquamous carcinoma (9), mucoepidermoid carcinoma (8), sarcomatoid carcinoma (3), Pleomorphic carcinoma (2), lymphepithelioma-like carcinoma (1), adenoid cystic carcinoma (1), and basaloid cell carcinoma (1)

**No information on the pathological differentiation status was available in 81 cases.

***No information on the T-stage was available in one case.

A comparative analysis of the TGase 2 expression and clinicopathologic parameters in the 429 NSCLC cases (Table 2) showed that TGase 2 levels were significantly higher in adenocarcinoma than in squamous cell carcinoma (p < 0.001), in females than in males (p < 0.001), and in nonsmokers than in smokers (p < 0.001). TGase 2 expression was also significantly correlated with T-stage (p = 0.037), but not with pathologic differentiation (p = 0.474) or nodal status (p = 0.414), as shown in Table 2.

**Figure 3:**
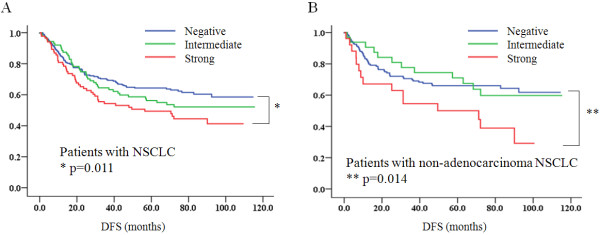
**Kaplan-Meier plot of DFS according to different levels of TGase 2 expression in patients in the entire NSCLC population (A) or in the non-adenocarcinoma subtype (B)**. For analysis of the immunohistochemical staining, the weak and intermediate TGase 2-expression groups were combined into an intermediate group. The DFS of the patients with strong TGase 2 expression (red) was significantly shorter (* and **) than that of the TGase 2-negative patients (blue) in both groups (x-axis: months after surgery; y-axis: DFS probability).

TGase 2 expression was significantly associated also with recurrence rate in the operable early-stage NSCLC cases examined (p = 0.005), as shown in Table 2. Notably, TGase 2 expression was significantly related to recurrence in the male (p = 0.011) and non-adenocarcinoma (p = 0.031) subgroups. By contrast, TGase 2 expression in the adenocarcinoma (p = 0.512) and female (p = 0.672) subgroups did not show any significant association with NSCLC recurrence. In a Kaplan-Meier plot of the total NSCLC patient population or of patients with the non-adenocarcinoma subtype, strong TGase 2 expression was correlated with shorter DFS (p = 0.011 for NSCLC, and p = 0.014 for the non-adenocarcinom subtype by log rank test) (Figure 3). Squamous cell carcinoma was the major histologic type in the non-adenocarcinoma subtype of NSCLC. But other histologic types, including large cell carcinoma, adenosquamous cell carcinoma, and mucoepidermoid carcinoma, also were included in the analysis (Table 2).

In a univariate analysis (Table 3), DFS was significantly related to strong TGase 2 expression (p = 0.011) as well as the clinicopathologic variables including histologic type (p = 0.044), T-stage (p = 0.044), and N-stage (p < 0.001). In a multivariate analysis considering T-stage, N-stage, and histological subtype as co-variables using Cox's proportional hazard regression model, TGase 2 expression was significantly related to shorter DFS (p = 0.029, and HR = 1.554, Table 3).

**Table 3:** Univariate and multivariate analyses of relationship between DSF and TGase 2 expression or clinicopathologic variables by Cox proportional hazard regression model (n = 429)

	Univariate analysis			Multivariate analysis		
	
	Hazard ratio	95% CI	p-value	Hazard ratio	95% CI	p-value
Age	1.001	0.986-1.016	0.889			

Gender						
Male	1	1				
Female	1.107	0.794-1.542	0.549			

Histology			0.063			0.072
Adenocarcinoma	1	1		1		
Squamous carcinoma	0.718	0.521-0.991	**0.044**	0.701	0.487-1.008	0.056
Others	1.200	0.705-2.042	0.501	1.214	0.710-2.076	0.478

Differentiation			0.172			
Well	1	1				
Moderate	1.424	0.862-2.354	0.168			
Poorly	1.699	0.977-2.955	0.061			

Smoking status			0.165			
Non-smoker	1					
Ex-smoker	1.060	0.720-1.560	0.769			
Current smoker	0.766	0.535-1.096	0.144			

T-Stage			**0.044**			**0.012**
1	1	1		1	1	
2	1.323	1 0.879-1.992	0.180	1.463	0.962-2.224	0.069
3-4	1.898	1.143-3.150	**0.013**	2.209	1.308-3.731	**0.003**

N-Stage			**<0.001**			**<0.001**
0	1	1		1	1	
1	1.613	1.116-2.330	**0.011**	1.625	1.118-2.363	**0.011**
2-3	2.121	1.459-3.084	**<0.001**	2.074	1.422-3.025	**<0.001**

TGase 2 expression			**0.039**			0.086
Negative	1	1		1	1	
Intermediate	1.230	0.846-1.789	0.277	1.111	0.752-1.642	0.597
Strong	1.595	1.112-2.287	**0.011**	1.554	1.046-2.311	**0.029**

In the multivariate analysis, variables including histology, T-stage and N-stage (those showing p < 0.10 in the univariate analysis) were considered.

In the non-adenocarcinoma subtype, strong TGase 2 expression also was significantly associated with shorter DFS (p = 0.016, Table 1). Moreover, a multivariate analysis on the non-adenocarcinoma subtype considering histologic type, differentiation, and clinical stage as co-variables, also showed strong TGase 2 expression to be significantly correlated with shorter DFS (p = 0.030, HR = 2.184), as indicated in Additional File 3, Table S1. In the male NSCLC subgroups, strong TGase 2 expression was significantly related to shorter DFS (p = 0.024, data not shown), but the association, according to the multivariate analysis, was not significant.

## Discussion

Previous reports [5,9] have posited cisplatin- and doxorubicin-resistance roles for TGase 2 in NSCLC cells. In the present study, we showed that TGase 2 down-regulation in lung cancer cells inhibited their invasive and migratory properties via the regulation of MMP-9. In immunohistochemical staining with Korean early-stage NSCLC tissues, strong TGase 2-positive cases had significantly higher rates of recurrence than TGase 2-negative cases (p = 0.005), and shorter DFS (p = 0.011). Moreover, this correlation was statistically significant in a multivariate analysis (p = 0.029), suggesting that TGase 2 is an independent prognostic marker for early-stage NSCLC survival.

The role of TGase 2 as an independent prognostic factor in surgically curative early-stage cancers appears to be evident in NSCLC. However, the role of TGase 2 in whole NSCLC cases is not as strong as in ovarian cancer [11]. In ovarian cancer, the correlation between survival and TGase 2 expression was shown to be statistically significant in a study of only 93 patients [11]. When we analyzed an identical number of the present NSCLC population, the association between TGase 2 expression and survival was not statistically significant. This suggests that TGase 2 might have a correlation with survival only in some subgroup(s) of NSCLC. To address this, we analyzed the correlation in different NSCLC subpopulations according to histologic type, gender, and smoking history. In the analysis, the association was significant in the male subgroup and non-adenocarcinoma subtype, but not in the female subgroup or in the adenocarcinoma subtype. In a multivariate analysis of the non-adenocarcinoma subtype, a significant association between TGase 2 expression and DFS was found, suggesting that TGase 2 expression is a prognostic marker only in the non-adenocarcinoma subtype including squamous cell carcinoma.

Until recently, the histologic subtyping of NSCLC was not clinically or therapeutically important. However, the adenocarcinoma and non-adenocarcinoma subtypes of NSCLC were suspected as separate entities, owing to the preferential responses to recently developed agents in adenocarcinoma [1-3]. This has encouraged research of new targets in adenocarcinoma NSCLC, and revisits the importance of subtyping not-otherwise-specified bronchial biopsy specimens [15]. However, the molecular targets in the non-adenocarcinoma subtype have not been well studied, and new targeted drug development in this subtype has been quite limited. The difference in therapeutic response might be related to the origin of cell types and to the subsequent molecular differences in the course of cancer progression. In this context, our finding of a specific association between TGase 2 expression and DFS in the non-adenocarcinoma subtype might point to TGase 2 as a molecular target in the non-adenocarcinoma subtype of NSCLC, which includes squamous cell carcinoma.

The enhanced migratory or invasive properties of TGase 2 expression might be related to the high binding affinity of TGase 2 to cell adhesion molecules such as fibronectin and integrin [16-19]. The interaction between TGase 2 and cell adhesion molecules in relation to invasion and metastasis has been further investigated in breast and ovarian cancers [12,13]. TGase 2-mediated induction of EMT along with the over-expression of various transcription repressors such as *Snail*, *Zeb1*, *Zeb2 *and *Twist1 *also has been reported, suggesting that TGase 2 is a key molecule involved in metastasis via EMT [20]. However, in the present study, TGase 2 down-regulation in a squamous lung cancer cell line did not afftect the level of EMT markers, suggesting the induction of NSCLC invasion might not be via EMT pathway. In addition, high expression of TGase 2 in NSCLC can enhance the apoptotic threshold via the induction of NF-κB, enabling survival of migrating cancer cells in the face of harsh micro-environmental stresses during the metastatic process. In the process, intracellular TGase 2 cross-links the inhibitory subunit α of nuclear factor (I-κBα), thus releasing NF-κB from its binding with I-κBα [21,22]. Although the invasive property was shown to be related to TGase 2 expression in squamous NSCLC cell lines via the regulation of MMP-9 in the present study, the reason that TGase 2 expression has an impact on survival or recurrence only in the non-adenocarcinoma subtype remains to be revealed.

## Conclusion

This study showed a significant association between TGase 2 expression and recurrence or shorter DFS, implicating TGase 2 as a prognostic marker in non-adenocarcinoma NSCLC. Recurrence or shorter survival in cases with higher TGase 2 expression might be related to the fact that TGase 2 over-expression enhances the invasive and migratory properties of NSCLC cells. The present study might also suggest that TGase 2 is a molecular target for the treatment of the non-adenocarcinoma subtype, which might be of importance with regard to the clinical application of TGase 2 inhibitor therapy.

## Competing interests

The authors declare that they have no competing interests.

## Authors' contributions

CMC and SJJ made the tissue microarrays, analyzed the immunohistochemical staining data, collected the clinical data and contributed to the writing of the manuscript. SYP, DSK and KSP performed the cell culture, *in vitro *invasion and migration assays. SYP, DSK and HKK performed immunohistochemical staining. JHJ and HYK participated in collecting the clinical data. JHJ and BHN analyzed the statistical data. SYK participated in designing the study and in writing the manuscript. KMH participated in designing and coordinating the study, and in writing the manuscript. All of the authors read and approved the final manuscript. KTORG supported this project as one of the collaborative projects.

## Supplementary Material

Additional File 1 **Figure S1. TGase 2 and NF-κB expression in NSCLC cells**. H1703 showed higher level of *TGM2 *or TGase 2 than HCC-95, and down-regulation of *TGM2 *in the H1703 cell decreased NF-κB activity. Click here for file

Additional file 2 **Figure S2. The relationship between TGase 2 expression and MMP-9 activity in squamous lung cancer cell lines**. Relationship between TGase 2 expression and matrix metalloproteinases (MMPs) or epithelial mesenchymal transition (EMT) markers was tested, and it was shown that only MMP-9 has a positive correlation with TGase 2 expression, suggesting that TGase 2's role in invasion and migration might be via the regulation of MMP-9. Click here for file

Additional file 3 **Table S1. Univariate and multivariate analyses of relationship between DFS and clinicopathologic variables or TGase 2 expression by Cox proportional hazard regression model in non-adenocarcinoma patients with NSCLC**. A multivariate analysis on the non-adenocarcinoma subtype considering histologic type, differentiation, and clinical stage as co-variables, also showed strong TGase 2 expression to be significantly correlated with shorter DFS. Click here for file
