# Supplementary material for: Transglutaminase 2 as an independent prognostic marker for survival of patients with non-adenocarcinoma subtype of non-small cell lung cancer
Source: Mol Cancer. 2011 Sep 24;10:119. doi: 10.1186/1476-4598-10-119 (PMC3196741; doi:10.1186/1476-4598-10-119)
Supplement: Additional File 1 — Figure S1. TGase 2 and NF-κB expression in NSCLC cells. H1703 showed higher level of TGM2 or TGase 2 than HCC-95, and down-regulation of TGM2 in the H1703 cell decreased NF-κB activity. [file 1476-4598-10-119-S1.DOC]

**
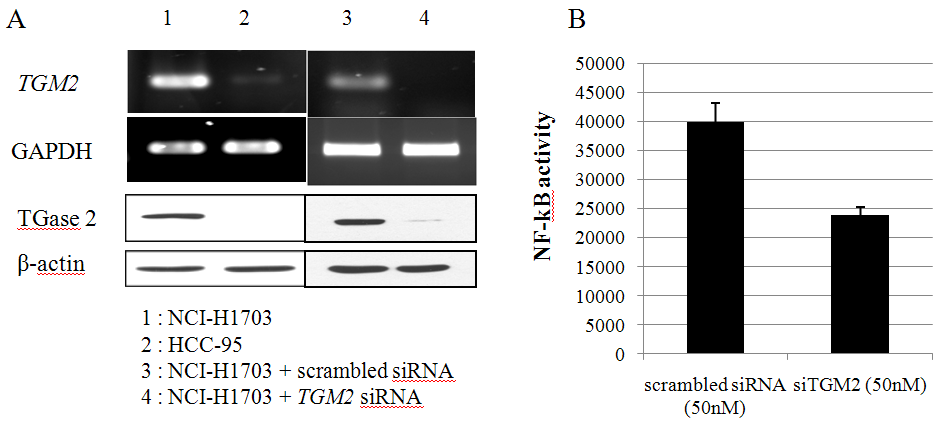
**

**Figure S1.** TGase 2 and NF-κB expression in NSCLC cells. A. The levels of TGase 2 protein or *TGM2* in the NSCLC cell lines H1703 and HCC-95 were determined by both reverse transcription polymerase chain reaction and Western blotting (lane 1 and 2). *GAPDH* and β-Actin were used as mRNA and protein controls. After the treatment of either *TGM2* siRNA or scramble siRNA in the lung cancer cell line H1703, changes of TGase 2 or *TGM2* expression were determined by reverse transcription polymerase chain reaction and Western blotting (lane 3 and 4). *GAPDH* and β-Actin were used as the mRNA and protein controls. B. NF-κB activity was measured by SEAP reporter assay after scramble siRNA or *TGM2* siRNA treatment in H1703 for either 24hr or 48 hr incubation.
