# Supplementary material for: Transglutaminase 2 as an independent prognostic marker for survival of patients with non-adenocarcinoma subtype of non-small cell lung cancer
Source: Mol Cancer. 2011 Sep 24;10:119. doi: 10.1186/1476-4598-10-119 (PMC3196741; doi:10.1186/1476-4598-10-119)
Supplement: Additional file 2 — Figure S2. The relationship between TGase 2 expression and MMP-9 activity in squamous lung cancer cell lines. Relationship between TGase 2 expression and matrix metalloproteinases (MMPs) or epithelial mesenchymal transition (EMT) markers was tested, and it was shown that only MMP-9 has a positive correlation with TGase 2 expression, suggesting that TGase 2's role in invasion and migration might be via the regulation of MMP-9. [file 1476-4598-10-119-S2.DOC]

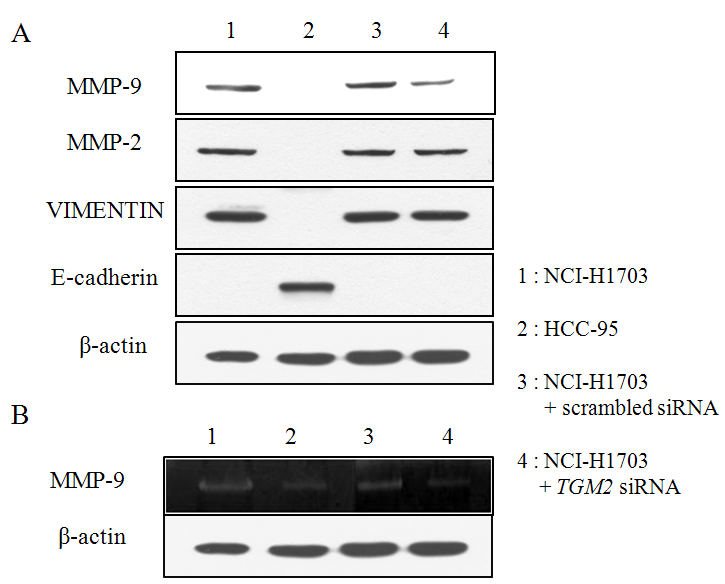


**Figure S2**. The relationship between TGase 2 expression and MMP-9 activity in squamous lung cancer cell lines. A. Relationship between TGase 2 expression and matrix metalloproteinases (MMPs) or epithelial mesenchymal transition (EMT) markers was tested. The levels of MMP-9 and MMP-2 were higher in H1703 cells with a higher level of TGase 2 than in HCC-95 cells. Whereas the expression of MMP-9 was reduced by down-regulation of TGase 2, the MMP-2 expression was not changed. EMT markers such as Vimentin and E-cadherin did not show significant differences by down-regulation of TGase 2. B. Decreased MMP-9 activity by down-regulation of TGase 2 was shown in gelatin zymography. MMP-9 activity was higher in the NCI-H1703 cells (lane 1) than in the HCC-95 cells (lane 2), but was reduced, by down-regulation of TGase 2 in the NCI-H1703 cells (lane 4) relative to the scramble siRNA-treated cells (lane 3).
