# Supplementary material for: Transglutaminase 2 as an independent prognostic marker for survival of patients with non-adenocarcinoma subtype of non-small cell lung cancer
Source: Mol Cancer. 2011 Sep 24;10:119. doi: 10.1186/1476-4598-10-119 (PMC3196741; doi:10.1186/1476-4598-10-119)
Supplement: Additional file 3 — Table S1. Univariate and multivariate analyses of relationship between DFS and clinicopathologic variables or TGase 2 expression by Cox proportional hazard regression model in non-adenocarcinoma patients with NSCLC. A multivariate analysis on the non-adenocarcinoma subtype considering histologic type, differentiation, and clinical stage as co-variables, also showed strong TGase 2 expression to be significantly correlated with shorter DFS. [file 1476-4598-10-119-S3.DOC]

Table S1. Univariate and multivariate analyses of relationship between DFS and clinicopathologic variables or TGase 2 expression by Cox proportional hazard regression model in non-adenocarcinoma patients with NSCLC (n=236). In the multivariate analysis, variables including histology, differentiation and clinical stage (those showing p <0.10 in the univariate analysis) were considered.

|  | Univariate analysis | | | Multivariate analysis | | |
| --- | --- | --- | --- | --- | --- | --- |
|  | Hazard ratio | 95% CI | p-value | Hazard ratio | 95% CI | p-value |
| Age | 1.003 | 0.983 to 1.024 | 0.766 |  |  |  |
| Gender  Male  Female | 1  1.168 | 1  0.602–2.268 | 0.646 |  |  |  |
| Histology  Squamous  Others | 1  1.638 | 1  0.946–2.836 | 0.078 | 1  2.780 | 0.961–8.040 | 0.059 |
| Differentiation  Well  Moderate  Poorly | 1  0.773  1.696 | 1  0.358–1.667  0.748–3.843 | **0.018**  0.511  0.206 |  |  |  |
| Smoking status  Non-smoker  Ex-smoker  Current smoker | 1  1.207  0.749 | 0.603–2.417  0.383–1.462 | 0.147  0.596  0.397 |  |  |  |
| Clinical Stage*  1  2  3 | 1  1.682  **1.792** | 1  0.992–2.851  **1.044–3.076** | 0.057  0.054  **0.034** | 1  1.187  1.632 | 1  0.639–2.207  0.900 to 2.959 | 0.269  0.588  0.106 |
| TGase 2 expression  Negative  Intermediate  Strong | 1  0.999  **2.033** | 1  0.533–1.871  **1.144–3.612** | **0.047**  0.996  **0.016** | 1  0.937  **2.184** | 1  0.451–1.944  **1.078–4.424** | 0.080  0.861  **0.030** |

*A multivariate analysis for the N-stage instead of the clinical stage yielded a similar result (P=0.032, HR=2.193 with 95% CI of 1.070-4.495).
